# *Vd*PLP, A Patatin-Like Phospholipase in *Verticillium dahliae*, Is Involved in Cell Wall Integrity and Required for Pathogenicity

**DOI:** 10.3390/genes9030162

**Published:** 2018-03-13

**Authors:** Xiliang Qi, Xiaokang Li, Huiming Guo, Ning Guo, Hongmei Cheng

**Affiliations:** 1 Biotechnology Research Institute, Chinese Academy of Agricultural Sciences, Beijing 100081, China; qiliangxi@163.com (X.Q.); lixiaokang2016@163.com (X.L.); guohuiming@caas.cn (H.G.); 2 Zhengzhou Fruit Research Institute, Chinese Academy of Agricultural Sciences, Zhengzhou 450009, China; 3 School of Life Sciences, Anhui Agricultural University, Hefei 230036, China; Guoning@ahau.edu.cn

**Keywords:** *Verticillium dahliae*, patatin-like phospholipases, cell wall integrity, pathogenicity

## Abstract

The soil-borne ascomycete fungus *Verticillium dahliae* causes vascular wilt disease and can seriously diminish the yield and quality of important crops. Functional analysis of growth- and pathogenicity-related genes is essential for revealing the pathogenic molecular mechanism of *V. dahliae*. Phospholipase is an important virulence factor in fungi that hydrolyzes phospholipids into fatty acid and other lipophilic substances and is involved in hyphal development. Thus far, only a few *V. dahliae* phospholipases have been identified, and their involvement in *V. dahliae* development and pathogenicity remains unknown. In this study, the function of the patatin-like phospholipase gene in *V. dahliae* (*VdPLP*, VDAG_00942) is characterized by generating gene knockout and complementary mutants. Vegetative growth and conidiation of *VdPLP* deletion mutants (*ΔVdPLP*) were significantly reduced compared with wild type and complementary strains, but more microsclerotia formed. The *ΔVdPLP* mutants were very sensitive to the cell-wall-perturbing agents: calcofluor white (CFW) and Congo red (CR). The transcriptional level of genes related to the cell wall integrity (CWI) pathway and chitin synthesis were downregulated, suggesting that *VdPLP* has a pivotal role in the CWI pathway and chitin synthesis in *V. dahliae*. *ΔVdPLP* strains were distinctly impaired in in their virulence and ability to colonize *Nicotiana benthamiana* roots. Our results demonstrate that *VdPLP* regulates hyphal growth and conidial production and is involved in stabilizing the cell wall, thus mediating the pathogenicity of *V*. *dahliae*.

## 1. Introduction

*Verticillium dahliae*, a soil-borne, filamentous fungal plant pathogen with wide distribution around the world, can attack many important crops such as cotton, soybean, potato, sugar beet and sunflower, causing significant crop losses annually [1,2]. It is a significant threat to cash-crop production. It also produces microsclerotia, dormant structures that can survive for many years in the soil and be induced to germinate by plant root exudates [3,4]. Hyphae colonize the plant root surface, and then form a narrow penetration peg that can penetrate the epidermal cells [5]. Hyphae grow intracellularly and intercellularly through the root cortex, enter the xylem vessels and colonize the xylem of the hypocotyl and leaves. Ultimately, water flow in the plant tissue is disrupted, causing the wilt symptoms, stunting, chlorosis and necrosis [4,6]. The molecular mechanisms underlying *V. dahliae* pathogenesis are complex and still not clear [4,5,6,7,8], so functional analyses of growth- and pathogenicity-related genes are crucial in developing new strategies to control *V*. *dahliae*.

Here, we focused on fungal phospholipases, which are involved in hyphal development, virulence and signaling. Phospholipases, key metabolic enzymes needed by all living organisms, hydrolyze phospholipids into fatty acids and lipophilic substances and are classified by the specific site of bond cleavage in the phospholipid substrates [9]. Phospholipase A enzymes hydrolyze the 1-acyl ester (PLA_1_) or the 2-acyl ester (PLA_2_) of phospholipids, and PLA enzyme actions produce free fatty acids and 2-acyl lysophospholipid (PLA_1_) or 1-acyl lysophospholipid (PLA_2_) [10,11]. Phospholipase B (PLB) enzymes possess hydrolytic activities that release sn-1 and sn-2 fatty acids from a phospholipid and PLBs catalyze the release of the remaining fatty acid linked to the lysophospholipid by lysophospholipase activity. Additionally, some PLBs can transfer a free fatty acid to a lysophospholipid and produce a phospholipid by transacylase activity [10,12,13]. Phospholipase C (PLC) catalyzes hydrolysis of phosphatidylinositol 4,5-bisphosphate (PIP2) to two intracellular messengers, inositol 1,4,5-triphosphate (IP3) and diacylglycerol (DAG) [10,14,15]. Phospholipase D (PLD) hydrolyzes phosphatidylcholine (PC) at second phosphodiester bond to yield phosphatidic acid (PA) and choline or ethanolamine. PLDs can also transfer the phosphatidyl moiety of the substrate of certain nucleophiles such as ethanol, which produce phosphatidylethanol (PEt) by the unique transphosphatidylation activity [9,10,16]. Lower phospholipase activities of pathogenic yeast fungi (*Candida albicans*, *Candida parapsilosis*, and *Saccharomyces cerevisiae*) reduce adherence to epithelial cells and are less lethal to mice [9,17]. During the development of *C. albicans* blastospores, phospholipase activities are concentrated at the growing tips [18]. PLC stimulates calcium release from intracellular stores and participates in hyphal extension at the growing tips in filamentous fungus *Neurospora crassa* [19]. In addition, phosphoinositide-specific PLC plays an important role in vegetative growth and cell wall regeneration in *Coprinopsis cinerea* [20].

Phospholipases are also related to signaling messengers to elicit stress tolerance and hosts immune responses in fungi [9,13,21]. The expression of phospholipases is regulated by persistent starvation [21,22]. PLA_2_ takes part in various physiological processes, such as phospholipids hydrolysis, signal transduction, and remodeling of cell membrane. Some secreted and membrane-bound PLA_2_ require Ca^2+^ to perform biological activities [10]. PLC catalyzes PIP2 to DAG and IP3. As second messengers, DAG and IP3 play an important role in the signal transduction cascade, DAG can activate protein kinase C (PKC) and IP3 can activate calcium channels [14,19]. In the symbiotic fungus *Tuber borchii*, TbSP1 is a novel Ca^2+^-activated phospholipase A_2_, which may take part in membrane remodeling and signal transduction during early stages of plant invasion [21]. PLCs cleave the glycerophosphate bond, thus are defined as phosphodiesterases [10]. PLDs hydrolyze PC to yield PA, which regulates the activation of the cAMP-specific phosphodiesterases (PDEs). PDEs and adenylyl cyclases (ACs) regulate the intracellular cAMP levels. cAMP signaling plays critical role in regulating multiple cellular responses [23,24]. In addition, high-affinity phosphodiesterase PdeH modulates intracellular cAMP levels, thereby regulates cAMP signaling, cellular development and pathogenicity of plant pathogenic fungus *Magnaporthe oryzae* [25,26,27]. MoMck1, as one of the components of the mitogen-activated protein kinase (MAPK), interacts with PdeH that regulates MAPK signaling pathway to regulate cell wall integrity [25,28].

The cell wall integrity (CWI) pathway can activate a compensatory salvage mechanism when the cell wall is under stress conditions [27,29]. MAPK pathways respond properly to extracellular stimuli or environmental conditions, and then regulate appropriate cellular responses [29,30,31]. In model yeast *S. cerevisiae*, cell wall stress is one of MAPK cascades that mediate the CWI pathway [30,31]. The MAP kinase I (Slt2/Mpk1) becomes activated under different cell wall stress conditions, such as hyposmolality, chitin-binding agents (Calcofluor White (CFW) and Congo Red (CR)) and oxidative stress. The major components of yeast cell wall are synthesized and modified under the stress [32,33,34]. The response mediated by Slt2/Mpk1 is defined as CWI pathway [31,33].

The patatin-like phospholipases (PLPs) was described as lipid acyl hydrolases. PLPs catalyze the cleavage of fatty acids from membrane lipids, thereby influence membranes remodeling [35,36]. PLPs have serine-aspartate dyads and α/β hydrolase fold, which is structurally similar to PLA_2_. Meanwhile, PLPs have been shown to exhibit a PLA_2_-like activity [36,37]. PLPs show difference in substrate specificity. PLPs have broader substrate preference, while cytosolic PLA_2_s show increased substrate specificity for arachidonic acid-containing lipids [36,37,38]. PLPs also do not contain a lid-like structure and are limited to interfacial activation [38]. Phospholipases have been studied in some plant pathogenic fungi, such as *Fusarium graminearum* [14] and *M. oryzae* [25,26,28]. The phospholipase C (FgPLC1) is considered to be closely related to regulation of development, stress responses and pathogenicity of *F. graminearum* [14]. cAMP phosphodiesterase PdeH regulates the intracellular cAMP levels, CWI and pathogenicity of *M. oryzae* [25,26,28], while the function of the patatin-like phospholipase (encoded by *VdPLP*, VDAG_00942) remains unclear in *V. dahliae*. The relations among *Vd*PLP, cell wall integrity and fungal virulence are unknown. In this study, to investigate the function of *Vd*PLP, we generated a deletion mutant (*ΔVdPLP*) of *V. dahliae* and characterized its growth and development, the stress tolerance and integrity of the cell wall, root penetration ability, and virulence in *Nicotiana benthamiana*.

## 2. Materials and Methods

### 2.1. Fungal Strains, Plant Material and Culture Conditions

*V. dahliae* strain 991 (*V991*, wild type (WT)) was kindly provided by Prof. Guiliang Jian from the Institute of Plant Protection, Chinese Academy of Agricultural Sciences (IPP, CAAS). *N. benthamiana* seedlings were planted in a growth chamber with 16 h of light/8 h of dark and 60–70% relative humidity at 23–25 °C.

### 2.2. Plasmid Construction and Fungal Transformation

**Figure 1:**
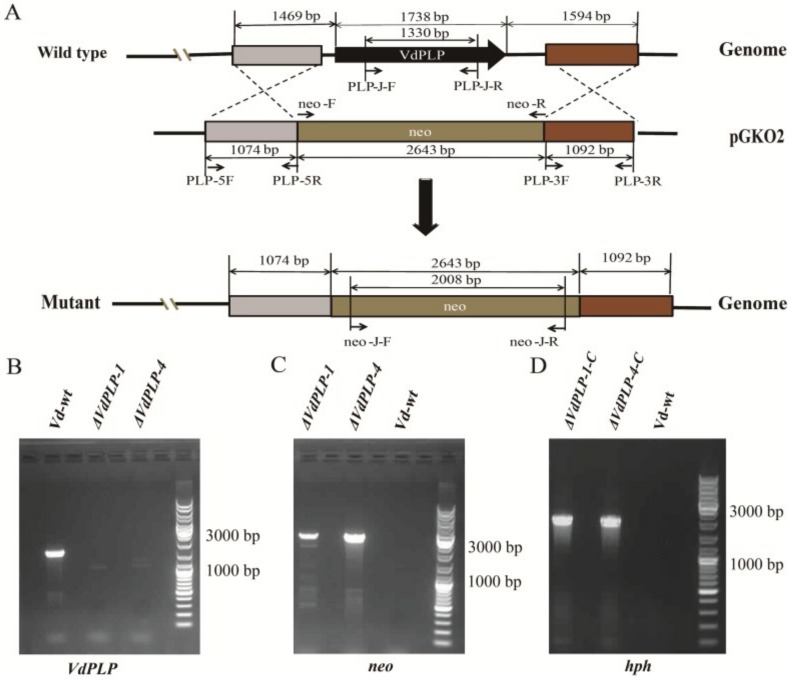
*VdPLP* gene disruption in *V. dahliae* and complementation of the *ΔVdPLP* strains (*ΔVdPLP-1* and *ΔVdPLP-4*). (**A**) Disruption scheme used for *VdPLP* in the wild type (wt) strain (Vd-wt); gene disruption was confirmed by PCR. Genomic DNA (gDNA) from the Vd-wt, *ΔVdPLP* and *ΔVdPLP-C* strains were used as templates for amplification with primer pairs VdPLP-J-F/VdPLP-J-R, neo-J-F/neo-J-R and hyg-F/hyg-R; (**B**) A 1.3-kb fragment of *VdPLP* was amplified from Vd-wt with primer pair VdPLP-J-F/VdPLP-J-R; (**C**) A 2.6 kb fragment of *neo* was amplified with primer pair neo-J-F/neo-J-R from the *ΔVdPLP* mutant strains; (**D**) Gene complementation of the *ΔVdPLP* strains (*ΔVdPLP-1-C* and *ΔVdPLP-4-C*) was confirmed by amplification of a 1.6 kb fragment of hygromycin B resistance gene (*hph)* in PCR with primer pair hyg-F/hyg-R.

*ΔVdPLP* mutants were obtained by a homologous recombination method. The *VdPLP* knockout plasmid pGKO-*VdPLP* (pGKO2 [*Eco*RI]::*VdPLP*-5′::*neo*::*VdPL*P-3′::pGKO2 [*Hind*III]) was constructed as in previous research [8,39]. The 2600 bp geneticin resistant cassette (*neo*) from pCAM-*neo* plasmid was amplified using primer pair neo-F/neo-R (Table 1). The 1074 bp upstream sequence of *VdPLP* were amplified from the *V. dahliae* genome with primer pairs VdPLP-5F/VdPLP-5R, and a 1092 bp downstream sequence with primer pairs VdPLP-3F/VdPLP-3R (Table 1). Vector pGKO2 [40] was linearized by double digestion with enzymes *Eco*RI and *Hind*III. Three PCR fragments were inserted into the corresponding position of linearized pGKO2 (Figure 1A) using In-Fusion enzyme (Clontech, Mountain View, CA, USA). 

For generating *VdPLP* complementary plasmid pCM-*Hyg*-*VdPLP*, the full-length complementary DNA (cDNA) fragment of *VdPLP*, the *TrpC* promoter and *Nos* terminator were amplified from plasmid pCH-GFP and primers C-VdPLP-F/C-VdPLP-R, C-TrpC-F/C-TrpC-R and C-Nos-F/C-Nos-R (Table 1). Plasmid pCM-*Hyg* carrying the *hygromycin B resistance* gene (*hph*) was linearized with restriction enzymes *Kpn*I and *Xba*I, and the fragments were inserted into plasmid pCM-*Hyg* digested with In Fusion enzyme.

Mutant strains were obtained through protoplast transformation [41]. Plasmids pGKO-*VdPLP*, pCM-*Hyg*-*VdPLP* and pCAM-*GFP* were used to transform protoplasts of the obtained mutants. *ΔVdPLP* mutant strains were generated by inserting plasmid pGKO-*VdPLP* into the wild type strain of *V. dahliae* (Vd-wt). Plasmid pCM-*Hyg*-*VdPLP* was introduced into *ΔVdPLP* strains to generate *VdPLP* complementary mutants (*ΔVdPLP-C*). GFP-tagged strains were generated by inserting plasmid pCAM-*GFP* carrying the *eGFP* gene and *hph* gene into Vd-wt and the *ΔVdPLP* strains.

### 2.3. Confirmation of VdPLP Disruption, Complementation of ΔVdPLP Strains and Screening for GFP-Tagged Strains

*VdPLP* knockout mutants were cultivated and selected on potato dextrose agar (PDA) plates supplied with geneticin (50 μg/mL). *ΔVdPLP-C* and GFP-tagged strains were selected in the presence of hygromycin B. After single-spore isolation, all isolates were grown in complete medium (CM, 6 g/L yeast extract, 6 g/L casein acid hydrolysate, 10 g/L sucrose) for DNA extraction. All isolates were further confirmed by genomic PCR with the specific primers. *VdPLP* was analyzed by genomic PCR with primer pair VdPLP-J-F/VdPLP-J-R (Table 1) for successful homologous recombination and with neo-J-F/neo-J-R for detecting the *neo* gene. *ΔVdPLP-C* mutants were selected by genomic PCR with primer pairs VdPLP-J-F/VdPLP-J-R and hyg-F/hyg-R (Table 1) for the *hph* gene. GFP-labeled strains were examined for the *hph* gene with primer pair hyg-F/hyg-R and for fluorescence with a confocal laser scanning microscope (LSCM; LSM 700, Zeiss, Jena, Germany) using 488 nm excitation wavelength and band-pass 500 to 550 nm emission filters.

### 2.4. Growth, Conidia Production and Microsclerotia Formation Assays

Vd-wt, *ΔVdPLP* and *ΔVdPLP-C* strains were cultured in CM, conidia of each strain were harvested and filtered through a sterile 40 μm cell strainer (Falcon, New York, NY, USA). Two microliters of 5 × 10^6^/mL conidial suspension of all strains were added to the center of plates of minimal medium (MM, 1.45 g/L KH_2_PO_4_; 2.05 g/L K_2_HPO_4_; 0.5 g/L NH_4_NO_3_; 0.01 g/L CaCl_2_; 0.6 g/L MgSO_4_·7H_2_O; 0.3 g/L NaCl; 0.5 mg/L ZnSO_4_·7H_2_O; 0.5 mg/L CuSO_4_·5H_2_O; 0.5 mg/L H_3_BO_3_; 0.25 g/L (NH_4_)_2_SO_4_; 0.5 mg/L MnSO_4_·H_2_O; 0.5 mg/L Na_2_MoO_4_·2H_2_O; 20 g/L agar) [6] amended with different carbon sources (10 g/L xylose; galactose; pectin; or starch; or 30 g/L sucrose). Colony diameter and morphology were recorded as described previously [9]. Vd-wt, *ΔVdPLP* and *ΔVdPLP-C* strains were cultured on Czapek-Dox agar (3.0 g/L NaNO_3_; 0.5 g/L MgSO_4_·7H_2_O; 0.5 g/L KCl; 0.01 g/L FeSO_4_·7H_2_O; 1.0 g/L K_2_HPO_4_; 15 g/L agar) plates to assay conidia production as described previously [39]. For microsclerotia formation tests, 10^6^/mL spores of Vd-wt, *ΔVdPLP* and *ΔVdPLP-C* strains were evenly spread on basal agar modified medium (BMM, 0.2 g/L NaNO_3_; 0.52 g/L KCl; 0.52 g/L MgSO_4_·7H_2_O; 1.52 g/L KH_2_PO_4_; 3 μmol/L thiamine; 0.1 μmol/L biotin; 5 g/L glucose; 15 g/L agar) plates, then incubated at 25 °C for 21 days. At seven-day intervals, microsclerotia on plates were counted [42]. Each strain was tested on 10 plates, and the assay was done three times.

### 2.5. Phenotype Assays Using Cell Wall-Disrupting Agents Calcoflour White and Congo Red

Susceptibility to cell wall-perturbing agents CFW and CR is generally tested to detect cell wall mutants of mycelial fungi [28,43]. A conidial suspension of each strain (10 µL of 5 × 10^6^/mL) was placed on the center of separate PDA agar plates containing 50 mg/mL CFW or 100 mg/mL CR, or without CR or CFW. Colony phenotypes were observed seven days after inoculation. The experiment was performed three times independently, using 10 plates per strain per treatment.

### 2.6. Quantitative Real-Time PCR Reaction

Cell wall reinforcement and repair in response to cell wall stress is activated by the CWI pathway, a basal cascade pathway triggered by transmembrane sensors in response to cell wall stress [44,45,46]. To examine the potential role of *Vd*PLP in the CWI pathway and chitin synthesis, we analyzed the transcription levels of genes encoding mitogen-activated protein kinase kinase (*VdMKK1*, VDAG_09823) and mitogen-activated protein kinase (*VdMK1*, VDAG_09461) for the CWI pathway and chitin synthesis genes *VdChi1* (VDAG_10179), *VdChi3* (VDAG_08591), *VdChi4* (VDAG_00419), *VdChi7* (VDAG_01790) in the Vd-wt, *ΔVdPLP* and *ΔVdPLP-C* strains, which were grown in liquid CM for 5 days. Total RNA was extracted from each strain using the RNA miniprep kit (Axygen, Union City, CA, USA) according to the user’s guide. cDNAs were synthesized with the TOYOBO RT Kit (TOYOBO, Osaka, Japan). The quantitative real-time PCR (qRT-PCR) reactions were performed with the SYBR Fast qPCR kit (KAPA Biosystems, Boston, MA, USA). Specific primers for different genes were designed (Table 1). The constitutively expressed β-tubulin gene (DQ266153) was used for normalization and amplified with primers VdBt-F/VdBt-R (Table 1). The qRT-PCR reactions were performed in an ABI7500 Fast PCR thermocycler (Applied Biosystems, Foster City, CA, USA). The experiment was repeated two times.

### 2.7. Penetration Ability Assay

Penetration was assayed as described previously [47,48]. About 20 µL of 2 × 10^6^/mL conidial suspension of each strain were placed on cellophane laid on PDA plates, then incubated at 25 °C. After 3 days, the cellophane was removed from the plates, which were then incubated for 7 more days at 25 °C, and hyphae were observed. This assay was done three times, with five plates per strain each time.

For microscopically observing initial infection, roots of *N. benthamiana* seedlings with 6–7 true leaves were inoculated with GFP-labeled *Vd-GFP* or *ΔVdPLP-GFP* strains [8]. After 3 days, the roots were vertically cut and observed on temporary slides with LSCM.

### 2.8. Pathogenicity Assays, Microscopic Observation of Initial Infection and Fungal Biomass Quantification

For pathogenicity assay experiments, roots of 10 *N. benthamiana* plants were inoculated with conidia of strain Vd-wt, *ΔVdPLP* or *ΔVdPLP-C* as previously described [39]. The disease symptoms were recorded at 8, 10, and 12 days post inoculation (dpi), and the mean of the disease grade was calculated as an indicator of the severity of the disease. Wilt symptoms were classified into five grades: 0, no disease symptoms; 1, wilting of fewer than two leaves; 2, wilting of three to five leaves; 3, wilting of more than five leaves; and 4, death or near death of plants [39,49].

Colonization ability of strains was assessed by quantifying the fungal biomass in *N*. *benthamiana* using qRT-PCR. DNA was extracted from infected plant stems using the DNAsecure Plant Kit (TIANGEN, Beijing, China). The *actin* gene of *N. benthamiana* was selected as an internal standard [8] and amplified with primer pair Nb-actin-F/Nb-actin-R (Table 1). ITS1 and ITS2 regions of the ribosomal RNA genes (Z29511) of *V. dahliae* were amplified with primer pair Vd-F/Vd-R (Table 1), which was used to quantify the fungal DNA in the mixed DNA samples. All qRT-PCR reactions were done as previously reported [39].

### 2.9. Statistical Analysis

Significant difference of data among the groups was analyzed using Duncan’s multiple range test (*p*-value < 0.05) and SPSS Statistics 17.0 software (SPSS, Chicago, IL, USA).

## 3. Results

### 3.1. Deletion and Complementation of VdPLP in *V. dahliae*

The *VdPLP* gene was replaced by the *neo* cassette in the Vd-wt strain. With the targeted gene replacement, geneticin resistance was generated. Transformations were confirmed by genomic PCR; homologous transformation events had occurred in 3 of 30 independent transformants of the Vd-wt strain. *VdPLP* deletion mutants (*ΔVdPLP-1* and *ΔVdPLP-4*) were randomly selected for further functional analysis (Figure 1B,C). For complementation, the In-fusion-resulting plasmid was introduced into *ΔVdPLP-1* and *ΔVdPLP-4* strains by protoplast transformation (Figure 1D), resulting in complementary strains *ΔVdPLP-1-C* and *ΔVdPLP-4-C*.

### 3.2. Radial Growth of Mycelia was Significantly Reduced in ΔVdPLP Mutants

**Figure 2:**
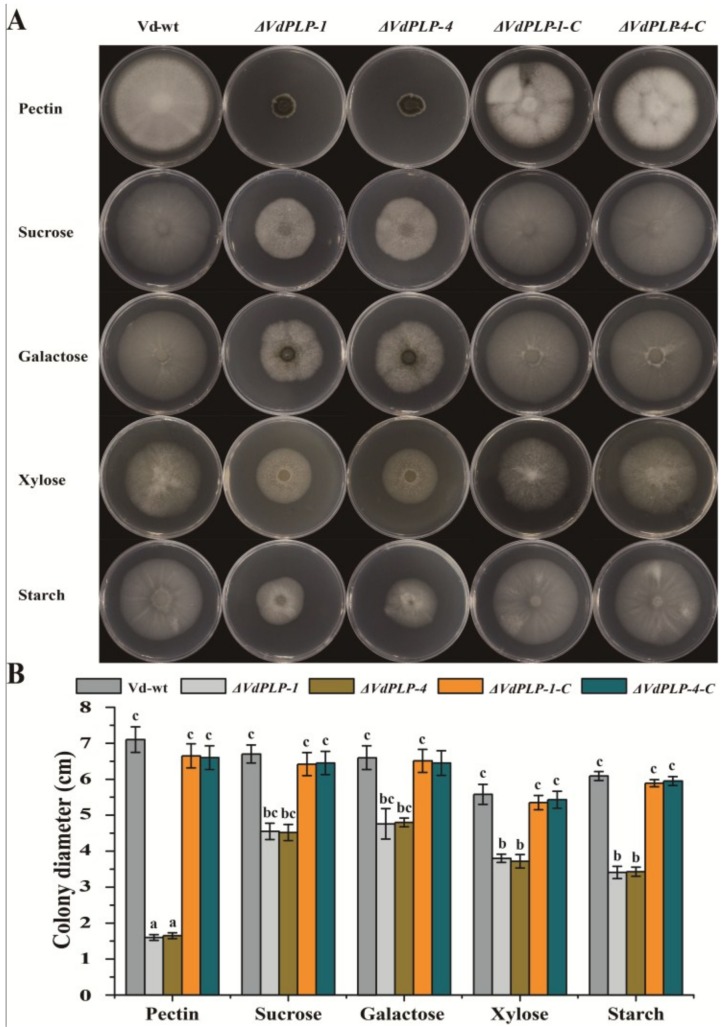
Mycelial growth of Vd-wt, *ΔVdPLP* (*ΔVdPLP-1* and *ΔVdPLP-4*) strains and complementary *ΔVdPLP* strains (*ΔVdPLP-1-C* and *ΔVdPLP-4-C*) strains after 15 days on minimal medium (MM) agar with different carbon sources: (**A**) colony morphology; and (**B**) colony diameters. Values represent the mean ± standard deviation (SD) from three independent replicates, Different letters (a–c) above the bars represent the significant differences among the groups (*p*-value < 0.05) and the data were analyzed using Duncan’s multiple range test.

The effects of *VdPLP* disruption on hyphal growth were defined by growing Vd-wt and the mutants on MM agar with different carbon source (sucrose, pectin, starch, galactose or xylose) and comparing the phenotypes of each strain. Vegetative growth of *ΔVdPLP* mutants was severely retarded compared with that of Vd-wt, *ΔVdPLP-1-C* and *ΔVdPLP-4-C* strains, and colony diameter of the *ΔVdPLP* mutants was significantly smaller than that of Vd-wt, *ΔVdPLP-1-C* and *ΔVdPLP-4-C* strains (Figure 2B). These results suggested that the *VdPLP* contributes to the hyphal growth of *V*. *dahliae* on different carbon sources.

### 3.3. Conidiation by ΔVdPLP Mutants Decreased Substantially and Microsclerotia Formation Increased

**Figure 3:**
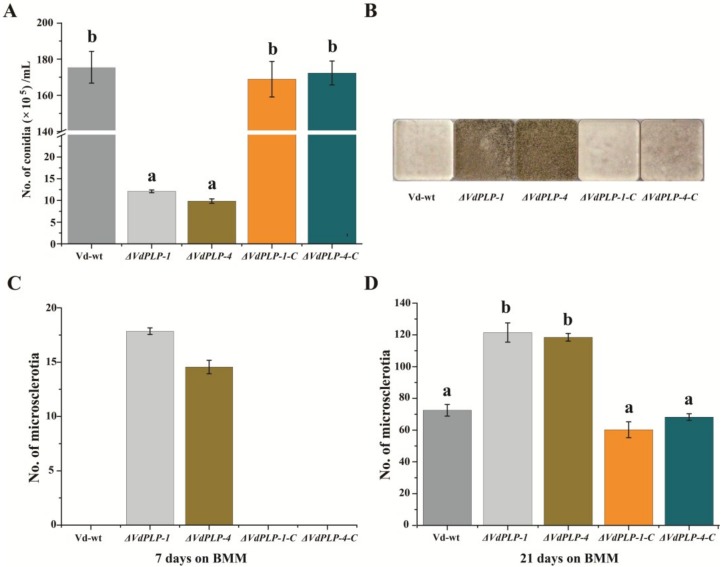
Deletion of *VdPLP* reduces conidial production and increases microsclerotial formation. (**A**) Number of conidia produced by Vd-wt, *ΔVdPLP* strains (*ΔVdPLP-1* and *ΔVdPLP-4*), and complementary *ΔVdPLP* strains (*ΔVdPLP-1-C* and *ΔVdPLP-4-C*) after 15 days on Czapek-Dox agar. (**B**) Colony color of Vd-wt, *ΔVdPLP* strains (*ΔVdPLP-1* and *ΔVdPLP-4*), and complementary *ΔVdPLP* mutant (*ΔVdPLP-1-C* and *ΔVdPLP-4-C*) strains after 21 days on basal agar modified medium (BMM) agar. Mean (±SD) number of microsclerotia after: seven days on BMM agar (**C**); and 21 days on BMM agar (**D**). Three independent replicates were done; Different letters (a–b) above the bars represent the significant differences among the groups and were analyzed with Duncan’s multiple range test (*p*-value < 0.05).

To check whether the deletion of *VdPLP* affects conidiation and microsclerotia formation, each strain was cultured on Czapek-Dox and BMM plates to enumerate conidia and microsclerotia after 15 days. Mutants *ΔVdPLP-1* and *ΔVdPLP-4* produced significantly fewer conidia on Czapek-Dox than did the Vd-wt and complementation strains (Figure 3A). In addition, the *ΔVdPLP* mutant strains accumulated more melanin on BMM, while Vd-wt, *ΔVdPLP-1-C* and *ΔVdPLP-4-C* strains were light-colored and produced no melanin on BMM (Figure 3B). On BMM, the *ΔVdPLP* strains formed microsclerotia earlier and in greater numbers than did the Vd-wt and the complementation strains (Figure 3C,D). The results indicated that deletion of the *VdPLP* gene accelerated melanin and microsclerotia production in *V. dahliae.*

### ΔVdPLP Mutants Are Hypersensitive to Cell Wall-Perturbing Agents

**Figure 4:**
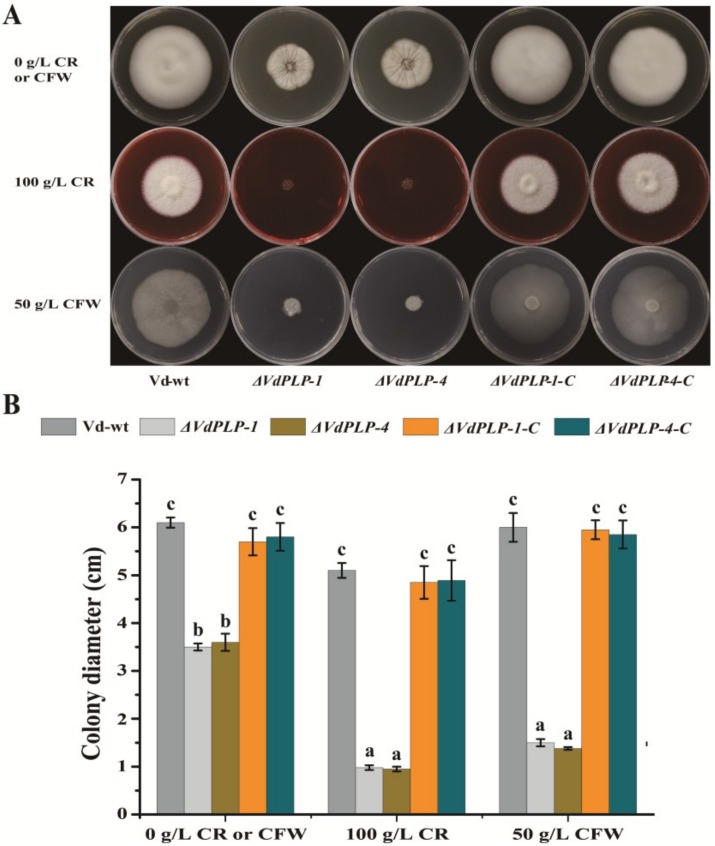
Effect of cell-wall-perturbing agents calcofluor white (CFW) and Congo red (CR) on growth of Vd-wt, *ΔVdPLP* (*ΔVdPLP-1* and *ΔVdPLP-4*) strains, and complementary *ΔVdPLP* (*ΔVdPLP-1-C* and *ΔVdPLP-4-C*) strains. (**A**) Colonies of Vd-wt, *ΔVdPLP* (*ΔVdPLP-1* and *ΔVdPLP-4*) mutant strains, and complementary *ΔVdPLP* mutant (*ΔVdPLP-1-C* and *ΔVdPLP-4-C*) strains after 15 days on potato dextrose agar (PDA) plates containing 100 g/L CR or 50 g/L CFW compared with controls on PDA at 25 °C. (**B**) Mean (± SD) colony diameter after 15 days on PDA plates with 100 g/L CR or 50 g/L CFW compared with controls on PDA. Three independent replicates were done; Different letters (a–c) above the bars represent the significant differences among the groups and were analyzed with the Duncan’s multiple range test (*p*-value < 0.05).

To determine whether the *VdPLP* gene was involved in maintaining cell wall integrity, we tested cell wall-perturbing agents CFW and CR for their effects on growth of the *ΔVdPLP* mutant strains. On PDA plates without CR or CFW, there was no significant difference between Vd-wt and the complementary *ΔVdPLP* strains in the colony diameter, but the colony diameter of *ΔVdPLP* strains showed nearly 50% reduction. On PDA plates containing 100 g/L CR or 50 g/L CFW, the colony diameter of *ΔVdPLP* was significantly smaller than that of Vd-wt and the complementary *ΔVdPLP* strains. Vd-wt and the complementary *ΔVdPLP* strains has no obvious growth defects on PDA plates with 100 g/L CR or 50 g/L CFW compared with growth on PDA without CR or CFW (Figure 4A,B). However, when 100 g/L CR or 50 g/L CFW was added to the medium, the colony diameter of *ΔVdPLP* strains was significantly smaller than that of the colony on PDA without CR. These results indicated that *ΔVdPLP* strains were more sensitive to CR and CFW than the wild type and complementary strains, and *VdPLP* was responsible for maintaining fungal cell wall integrity in *V. dahliae*.

### 3.5. Genes for the Calcofluor White Pathway and for Chitin Synthesis in the ΔVdPLP Mutant Were Downregulated

**Figure 5:**
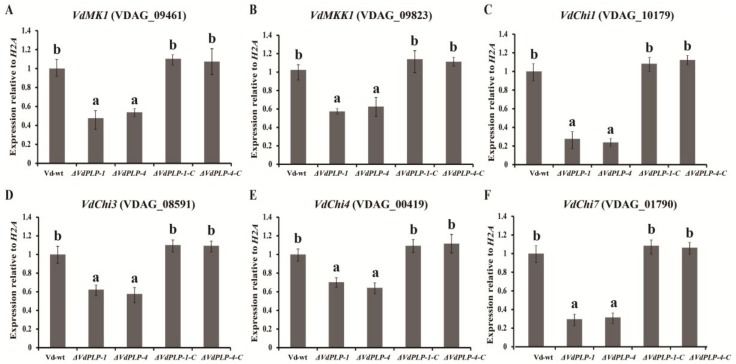
Expression of cell wall integrity (CWI) pathway genes (*VdMKK1* (VDAG_09823) and *VdMK1* (VDAG_09461)), and chitin synthesis genes (*VdChi1* (VDAG_10179), *VdChi3* (VDAG_08591), *VdChi4* (VDAG_00419) and *VdChi7* (VDAG_01790)) in the *ΔVdPLP* mutant strains (*ΔVdPLP-1* and *ΔVdPLP-4*) was lower than in the *V. dahliae* wild type (Vd-wt) strain and complementary *ΔVdPLP* strains (*ΔVdPLP-1-C* and *ΔVdPLP-4-C*). Total RNA was extracted from mycelia of seven-day-old Vd-wt, *ΔVdPLP* (*ΔVdPLP-1* and *ΔVdPLP-4*), and complementary *ΔVdPLP* (*ΔVdPLP-1-C* and *ΔVdPLP-4-C*) strains were grown in liquid complete medium (CM). Quantitative real-time reverse-transcription PCR was used to measure expression levels: *VdMKK1* (**A**); *VdMK1* (**B**); *VdChi1* (**C**); *VdChi3* (**D**); *VdChi4* (**E**); and *VdChi7* (**F**). The β-tubulin gene was used as an internal standard. Values are means ± SD of three independent experiments performed in duplicates, significant differences are indicated by letters (a,b), and data were analyzed with the Duncan’s multiple range test (*p*-value < 0.05).

To further confirm that *VdPLP* contributes to the CWI pathway and chitin synthesis, we focused on the regulation of the CWI pathway by profiling transcript levels of the CWI pathway genes *VdMKK1* and *VdMK1* and chitin synthesis genes *VdChi1*, *VdChi3*, *VdChi4*, and *VdChi7*. In the *ΔVdPLP* mutant strains, transcription of *VdMKK1*, *VdMK1*, and the chitin synthesis genes all decreased compared with the wild type strain and complementary *ΔVdPLP* strains (Figure 5A–F). Downregulation of CWI and chitin synthesis genes indicated that *VdPLP* participates in the CWI pathway and chitin synthesis.

### 3.6. Penetration and Fungal Colonization of ΔVdPLP Were Impaired

**Figure 6:**
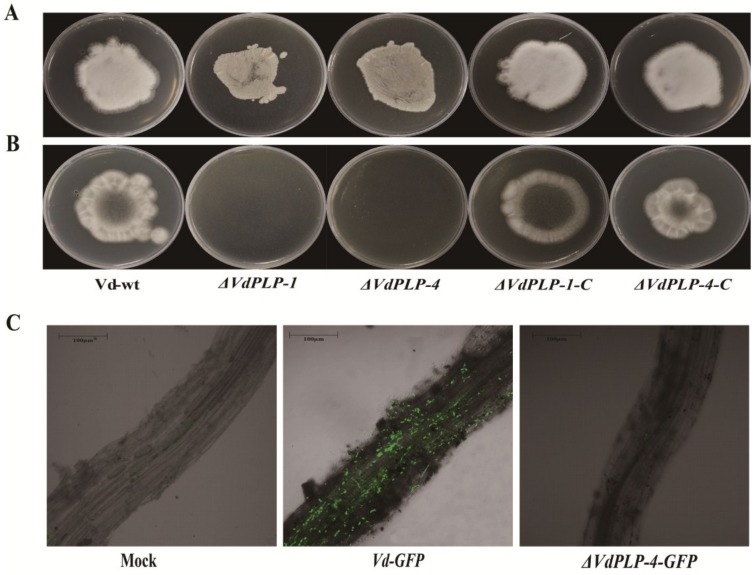
Fungal colonization and penetration assay: (**A**) Vd-wt, *ΔVdPLP* and *ΔVdPLP-C* strains grown on cellophane for three days after strains had penetrated a cellophane membrane on the agar plate and membranes were removed; (**B**) plates after incubation at 25 °C for seven days after cellophane were removed; and (**C**) fungal colonization of infected roots by the *ΔVdPLP-4-GFP* mutant strains, *Vd-GFP* strain, and sterile water (Mock), three days after inoculation.

To investigate possible reasons for the significant virulence defect of *ΔVdPLP* mutant strains, we analyzed penetration by the Vd-wt, *ΔVdPLP* and *ΔVdPLP-C* strains. After a conidial suspension of the respective strains was placed on cellophane membranes, Vd-wt and the *ΔVdPLP-C* strains penetrated and colonized the membranes, but no hyphae of the *ΔVdPLP* mutant strains grew (Figure 6A,B). Thus, the deletion of *VdPLP* severely hindered the ability to penetrate the cellophane membrane. 

GFP-labeled strains *ΔVdPLP-4-GFP* and *Vd-GFP* constitutively express *GFP*; strong green fluorescence thus allows observation of fungal penetration and colonization of plant root tissues using LSCM. Strain *Vd-GFP* strain colonized *N. benthamiana* roots, and hyphae penetrated the epidermal and cortical cells. In contrast, few hyphae of the *ΔVdPLP-4-GFP* strain grew on the root, and the strain penetrated epidermal cells (Figure 6C). These phenomena indicate that *VdPLP* contributes to penetration and colonization by *V. dahliae*. 

### 3.7. VdPLP was Required for Fungal Pathogenicity

**Figure 7:**
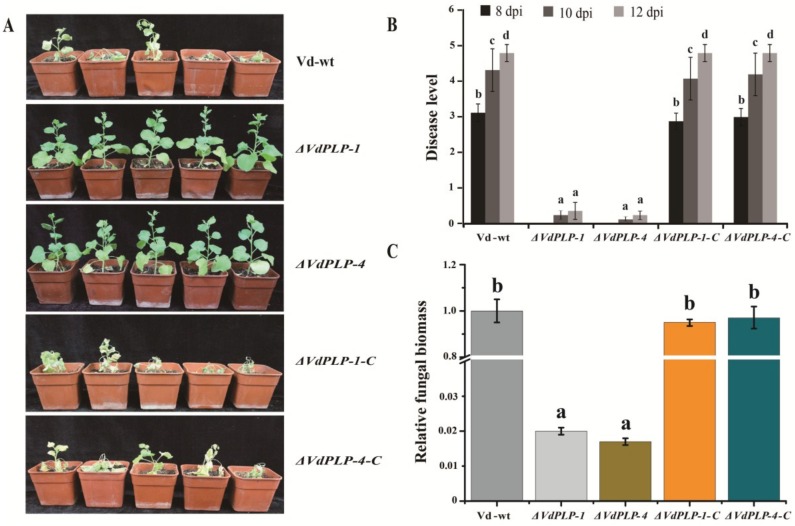
*ΔVdPLP* mutant strains were not virulent on *Nicotiana benthamiana* in pathogenicity assays. (**A**) Representative plants 12 days post inoculation (dpi) with Vd-wt, *ΔVdPLP* strains (*ΔVdPLP-1* and *ΔVdPLP-4*), or complementary *ΔVdPLP* strains (*ΔVdPLP-1-C* and *ΔVdPLP-4-C*); (**B**) Disease level on *N. benthamiana* after a 2-min root-dip in conidia of the Vd-wt, *ΔVdPLP* strains (*ΔVdPLP-2* and *ΔVdPLP-4*), and complementary *ΔVdPLP* mutant (*ΔVdPLP-1-C* and *ΔVdPLP-4-C*) strains at 8, 10, and 12 dpi. See methods for severity scale; (**C**) Relative quantification of fungal biomass in *N. benthamiana* stems inoculated with conidial suspension of Vd-wt, *ΔVdPLP* strains (*ΔVdPLP-1* and *ΔVdPLP-4*), and complementation *ΔVdPLP* strains (*ΔVdPLP-1-C* and *ΔVdPLP-4-C*) at 12 dpi. Values are means ± SD from three independent replicates. Significant differences are indicated by letters (a–d), Duncan’s multiple range test (*p*-value < 0.05) was used to analyze significant difference between each group.

To verify the role of *VdPLP* in fungal pathogenicity, we inoculated roots of *N. benthamiana* seedlings with a conidial suspension of Vd-wt, *ΔVdPLP*, or *ΔVdPLP-C* strains. As expected, at 8, 10, and 12 dpi, plants inoculated with either Vd-wt, or the complementary strains developed significant wilting, whereas only a few chlorotic leaves appeared on plants inoculated with *ΔVdPLP* strains. At 12 dpi, plants inoculated with Vd-wt or *ΔVdPLP-C* strains were stunted, chlorotic, wilting or dead. In contrast, the seedlings infected with *ΔVdPLP* mutants exhibited mild symptoms, with interveinal chlorosis on the bottom 1 or 2 leaves, but no necrosis (Figure 7A,B). Furthermore, when biomass was quantified, less fungal DNA was detected from infected plants inoculated with the *ΔVdPLP* strains than with Vd-wt or the *ΔVdPLP-C* strains (Figure 7C). The decrease in symptoms and biomass suggest that *VdPLP* disruption reduced the virulence of *V. dahliae*.

## 4. Discussion

Phospholipases also regulate vegetative growth signals in filamentous fungi [9,13,21,22,50]. The cAMP-specific PDEs can be regulated by some phospholipases, which adjust intracellular cAMP level. cAMP signaling participates in numerous intracellular activities [23,24]. For example, the constitutive activation of cAMP phosphodiesterase, which can activate the cAMP pathway, influences the biosynthesis and structure of the cell wall and membrane [51,52]. Fungal phospholipases affect blastospore development [18] and participate in hyphal extension in the filamentous fungi *N. crassa* [19], *C. cinerea* [20], and *M. oryzae* [25,28]. Due to inhibition of phosphodiesterase activity in *V. dahliae*, conidial production and virulence was reduced in G protein β subunit gene (*VGB*) mutants [53]. Similar phenotypic changes were observed in *VdPLP* deletion mutants. Hyphal growth of *ΔVdPLP* strains under different carbon sources conditions, and conidia production was drastically reduced compared with the wild type strain and complementation mutants, which suggested that impaired vegetative growth and conidial production caused by *VdPLP* deletion may be the reason for the virulence decline. 

Microsclerotia, melanized survival structures that can survive in the soil for more than ten years in the absence of host plants, play a critical role in the disease cycle of *V. dahliae*, but the molecular mechanism of their biogenesis is still not clear [54,55,56,57]. *VdPKAC1* mutants showed a decrease in fungal virulence and an increase in microsclerotial formation [58]. In contrast, microsclerotia production decreased by *V. dahliae* class II hydrophobin gene (*VDH1*) mutants [59], *Vayg1* deletion mutants [60], transmembrane mucin Msb (*VdMsb*) mutants [61] and MAPK mutants [62]. In the present laboratory study, *ΔVdPLP* mutants produced microsclerotia earlier and in greater numbers than did Vd-wt and the *ΔVdPLP-C* strains. Based on the above research, opposite results for mutants in different genes suggest that the molecular mechanisms of microsclerotia formation are complex. Regulatory genes such as *VdPKAC1*, *VDH1*, *Vayg1*, *VdMsb*, and dihydroxynaphthalene (DHN) melanin biosynthesis pathway [57] may regulate the microsclerotia biosynthesis. The loss of *VdPLP* likely activates microsclerotia production via participating in a signaling pathway, but the specific mechanisms of the transition from vegetative growth to resting structure development require further research.

The fungal cell wall plays a critical role in maintaining cell shape, protecting against physical injury, and mediating exchanges between pathogenic fungi and their hosts [62,63,64]. It also provides adhesive properties and protection against host defenses, which is imperative for pathogenicity in fungi [63,65,66]. PdeH are related to cell wall integrity, stress response and virulence in *M*. *oryzae* and *C*. *albicans* [25,28,50,51,52]. *Mo*PdeH targets *Mo*Mck1 of *M. oryzae*, one of the components of the MAPK cascade, and regulates cell wall integrity and virulence [28]. Integrity of the cell wall is critical for penetration of the host and thus for fungal virulence [67]. In this study, deletion of *VdPLP* generated *ΔVdPLP* mutants that were sensitive to cell wall-perturbing agents, which suggests *VdPLP* participates in stabilizing the cell wall under stress. Disruption of cell wall integrity in the *VdPLP* mutants was thus associated with the significantly reduced virulence of the *ΔVdPLP* mutants.

A small G protein, Rho1, is considered to be the master regulator of CWI signaling. Rho1 integrates signals from the cell surface and regulates many outputs involved in cell wall biogenesis. GTP-bound Rho1 associates with and activate the PKC1 [47]. Polyunsaturated fatty acids like arachidonic acid (AA) released by PLPs may regulate cellular signaling and influence the activity of protein kinases [35]. MAPK pathway can be regulated by protein kinases [30,31]. Thus, PLPs could influence the MAPK pathway and CWI pathway as well. In the citrus postharvest pathogen *Penicillium digitatum*, disruption of chitin synthase (*PdChsVII*) impaired CWI integrity, causing reduced virulence [68]. Class I chitin synthases reinforce cell wall integrity under a specific cell wall stress in *C. albicans* [69]. A myosin motor domain-chitin synthases (MMD-Chs) deletion impaired fungal resistance to host defense mechanisms and colonization by invasive hyphae, possibly due to a decrease in the structural integrity and permeability of the cell wall [70]. In our research, the expression of the MAPK pathway (*VdMK1* and *VdMKK1*) was diminished in the *ΔVdPLP* mutants. The CWI pathway and chitin synthesis genes were downregulated in the *ΔVdPLP* strains, suggesting that *VdPLP* has a critical role in regulating the CWI pathway and chitin synthesis in *V. dahliae*. Meanwhile, *ΔVdPLP* mutants cannot penetrate the cellophane membrane. GFP-labeled *ΔVdPLP* mutants showed impairment in colonization. The results indicate that *VdPLP* could regulate the MAPK pathway for regulating CWI pathway and chitin synthase and affect the cell wall integrity and virulence of *V. dahliae*.

Soil-borne *V*. *dahliae* must penetrate and colonize the root to cause disease. The colonization of *Arabidopsis thaliana* [71], cotton [6] and tomato [72] by *V. dahliae* GFP-labeled strains has been detailed. In our research, the same strategy was used for penetration ability comparison. When the penetration ability of GFP-tagged *ΔVdPLP* strains was compared with that of *Vd-GFP* strains, penetration of epidermal cells of the roots by hyphae of *ΔVdPLP-GFP* strains was severely lowered as was subsequent colonization. In addition, *ΔVdPLP* strains were impaired in penetration of cellophane, indicating impairment of an early phase of infection. *VdPLP* may regulate vegetative growth of *V. dahliae* in plant tissue and affect cell wall stability, and then impair pathogenicity further.

Deletion of *VdPLP* resulted in severe impairment of vegetative growth on various carbon sources and reduced conidia production. The *ΔVdPLP* mutants were also severely impaired in their ability to penetrate and colonize roots of *N. benthamiana*, as shown microscopically and by a decrease in biomass compared with the wild type. The qRT-PCR results suggest that *VdPLP* participates in the CWI pathway and chitin synthesis. These results revealed the defect of *ΔVdPLP* mutants to penetrate plant tissue, contributing to the impaired virulence. *VdPLP* may contribute to the full virulence of *V. dahliae* by mediating hyphal growth, the cell wall integrity and penetration process, which provides new insights into the molecular pathogenic mechanisms of *V*. *dahliae*.

## Figures and Tables

**Table 1:** List of primers used in this study.

Primer Name	Primer Sequence
neo-F	GTTTGCGGGCTGTCTTGACG
neo-R	TACCTGTGCATTCTGGGTAA
VdPLP-5F	GTACCCAATTCGAATTCCCAGCGGTTCGGGTAGTAGTAGA
VdPLP-5R	CAAGACAGCCCGCAAACGTATAACCCCGCGGAGCAGTAA
VdPLP-3F	CCCAGAATGCACAGGTAGACGCGCCACGACCTCAA
VdPLP-3R	ACGGTATCGATAAGCTTTGCGTGCGAACATACTCCTCAT
C-TrpC-F	TTGAAGGAGCATTTTTGGGC
C-TrpC-R	ATCGATGCTTGGGTAGAATAGGT
C-VdPLP-F	ATGCCTGTCAACGATATCCGTCT
C-VdPLP-R	CTATTCCTCGATCAGAGAGTAG
C-Nos-F	AGATGCCGACCGGGATCCACTT
C-Nos-R	TTATCTTTGCGAACCCAGGG
VdPLP-J-F	CTCGAGCGGGCCATCAAACA
VdPLP-J-R	GAGTAAGCCACCCATCTGTCCGTT
neo-J-F	GCGGTTCAGAAGCACCTCGA
neo-J-R	TATCTTTGCGAACCCAGG
VdMK1-F	CGCGCCCGAGATTATGCTGAG
VdMK1-R	CGTTGGGAGTACCGAGGATGTGAA
VdChi1-F	GCCGCCGCCTGGTCATC
VdChi1-R	CGGGGTAGAGGTCGGCATCA
VdChi3-F	GGTCGGCCCTTGGAGCAGTA
VdChi3-R	CCCTTGGCAGCCTTGATGTAGC
VdChi4-F	TACGGCAAGGTTTACTCGGGTCTC
VdChi4-R	CGGTTGCCAGGCTTCGTCTTAC
VdChi7-F	CATCCTCGGCGTCACAAAGTTCTA
VdChi7-R	GCTGCCGCTGCTGGAGGTA
VdBt-F	TTCCCCCGTCTCCACTTCTTCATG
VdBt-R	GACGAGATCGTTCATGTTGAACTC
Nb-actin-F	GGACCTTTATGGAAACATTGTGCTCAGT
Nb-actin-R	CCAAGATAGAACCTCCAATCCAGACAC
Vd-F	CCGCCGGTCCATCAGTCTCTCTGTTTATAC
Vd-R	CGCCTGCGGGACTCCGATGCGAGCTGTAAC
